# Development and Validation of a Risk-Prediction Nomogram for Chronic Low Back Pain Using a National Health Examination Survey: A Cross-Sectional Study

**DOI:** 10.3390/healthcare11040468

**Published:** 2023-02-06

**Authors:** Jung Guel Kim, Sang-Min Park, Ho-Joong Kim, Jin S. Yeom

**Affiliations:** Spine Center and Department of Orthopedic Surgery, Seoul National University College of Medicine and Seoul National University Bundang Hospital, Seongnam 13620, Republic of Korea

**Keywords:** low back pain, health survey, nomogram, prediction model, risk factor

## Abstract

Background: Several prognostic factors have been reported for chronic low back pain (CLBP). However, there are no studies on the prediction of CLBP development in the general population using a risk prediction model. This cross-sectional study aimed to develop and validate a risk prediction model for CLBP development in the general population, and to create a nomogram that can help a person at risk of developing CLBP to receive appropriate counseling on risk modification. Methods: Data on CLBP development, demographics, socioeconomic history, and comorbid health conditions of the participants were obtained through a nationally representative health examination and survey from 2007 to 2009. Prediction models for CLBP development were derived from a health survey on a random sample of 80% of the data and validated in the remaining 20%. After developing the risk prediction model for CLBP, the model was incorporated into a nomogram. Results: Data for 17,038 participants were analyzed, including 2693 with CLBP and 14,345 without CLBP. The selected risk factors included age, sex, occupation, education level, mid-intensity physical activity, depressive symptoms, and comorbidities. This model had good predictive performance in the validation dataset (concordance statistic = 0.7569, Hosmer–Lemeshow chi-square statistic = 12.10, *p* = 0.278). Based on our model, the findings indicated no significant differences between the observed and predicted probabilities. Conclusions: The risk prediction model presented by a nomogram, which is a score-based prediction system, can be incorporated into the clinical setting. Thus, our prediction model can help individuals at risk of developing CLBP to receive appropriate counseling on risk modification from primary physicians.

## 1. Background

Low back pain (LBP), one of the most common sources of pain from musculoskeletal disorders and a major public health problem that influences the functional status and quality of life in elderly people, and is reported in 70–80% of the general population at least once in their lifetime [1,2,3,4,5]. Patients with LBP may recover spontaneously, but some LBP patients will develop chronic LBP (CLBP) [6]. The prevalence grows linearly from the third decade of life to the age of 60 years, with a higher incidence among women [2,7]. In addition, a recent meta-analysis found that 21–68% of patients aged 60 or older had CLBP in the previous year, confirming the high incidence of CLBP among older adults [8]. Since the global population of people aged 60 or older is projected to quadruple by 2050 (reaching 2.1 billion), it is crucial to identify risk factors for CLBP in older adults in order to design and implement appropriate preventive and treatment methods for high-risk individuals [9].

The risk factors for LBP are multifactorial, complex, and remain poorly understood [10,11,12,13,14,15]. LBP prognosis is heavily affected by variables unrelated to the spine. The biopsychosocial model describes how psychological and social variables alter a person’s perception of symptoms [15]. Overemphasis on pain alone and reliance on merely mechanical nominal diagnosis might result in increased impairment. Clinicians should thus address all components (biomechanical, psychological, and psychosocial) of the condition when treating patients with LBP [16,17,18,19,20]. The most frequently identified risk factors for CLBP are greater pain intensity, higher body weight, carrying large loads at work, difficult working postures, and depression [7]. In addition, the direct predictors of chronicity were maladaptive behavior patterns, overall anxiety, functional restriction during the episode, smoking, and particularly physical work. According to a systematic study, various biomechanical, psychological, and psychosocial prognostic variables are relevant for low back pain chronicity. In these studies, CLBP was more closely associated with demographic, psychological, and occupational aspects than with the medical characteristics of the disorder itself [21,22,23,24,25,26]. However, these studies only evaluated the prognostic factors of patients who developed CLBP from patients with nonspecific LBP in hospital or workplace settings. In a community-based setting, it is important to identify risk factors for CLBP and predict the probability of those patients who are likely to develop CLBP. However, there have been no studies on the prediction probability of CLBP development in the general population using a risk prediction model.

Based on this background, the aims of this study were to develop and validate a risk prediction model for CLBP development, and to create a shared decision-making nomogram that can help a patient at risk of developing CLBP to receive appropriate counseling on risk modification using a nationally representative sample of Korean adults.

## 2. Methods

### 2.1. Study Design, Setting and Participants

**Figure 1:**
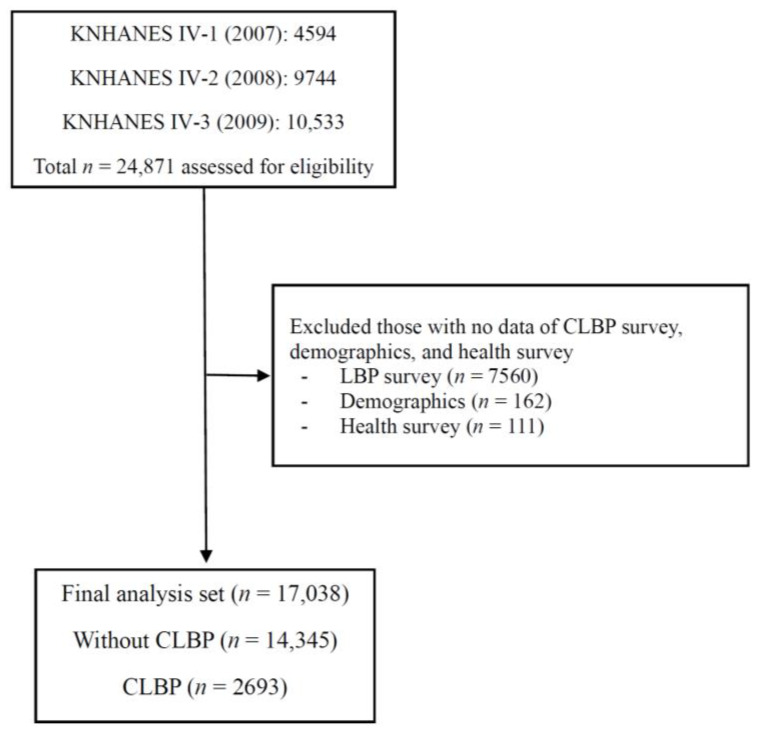
Flow diagram of the inclusion and exclusion of participants from the 2007, 2008 and 2009 Korea National Health and Nutrition Examination Surveys (KNHANES IV-1, IV-2 and IV-3). CLBP, chronic low back pain.

Data from versions IV-1, 2, and 3 of the Korea National Health and Nutrition Examination Survey (KNHANES) performed in 2007, 2008, and 2009 were analyzed. This survey was conducted annually since 1998 by the Korea Centers for Disease Control (KCDC). The Korean National Health and Nutrition Examination Survey (KNHANES) is a complicated, stratified, and multi-stage probability cluster survey in which participants were not selected at random from the Korean population. In addition to sample design errors, nonparticipation errors and nonresponse errors are also included in the KNHANES. In order to eliminate biases between estimators and population parameters, it is required to consider complicated sampling weights. KNHANES offers complicated sample weights for each participant, allowing data analysts to adjust for these biases. The KNHANES evaluates three aspects: health questionnaires, health and physical examinations, and nutrition questionnaires that are administered by experienced interviewers, registered nurses, and laboratory technicians [27,28]. KNHANES IV-1 (2007), IV-2 (2008), and IV-3 (2009) examinations and health surveys were completed by 4594, 9744, and 10,533 participants (24,871 participants in total), respectively. The present analysis was confined to 17,038 respondents aged 10–100 years who answered the CLBP examination survey and had no missing demographic and health questionnaire data (Figure 1).

### 2.2. Definitions of Chronic Low Back Pain

Participants who answered “yes” to all three questionnaires were defined as having CLBP: (1) “Have you ever had LBP at any point of your life?” (2) ‘Do you currently have LBP?’, and (3) “Have you complained of LBP for more than 90 days during the past year?”

### 2.3. Data Sources, Measurements and Variables

We analyzed participants’ demographics, socioeconomic status, comorbidities, and lifestyle habits through health interviews and examinations. All participants were asked whether they were previously diagnosed with major comorbidities by physicians, such as hypertension, diabetes mellitus, dyslipidemia, ischemic heart disease (myocardial infarction, angina), stroke, liver cirrhosis, asthma, chronic obstructive pulmonary disease, arthritis, and chronic kidney disease major cancers (lung, stomach, liver, colon, breast, prostate, or uterine cervical).

Age was categorized into groups. Body mass index (BMI) was calculated as body weight (kg) divided by height (m) squared and categorized into the following: underweight (<18.5 kg/m^2^), normal weight (18.5–24.9 kg/m^2^), and obese (≥25.0 kg/m^2^). The smoking status was determined as either non/ex-smokers or current smokers. Alcohol consumption was identified as none or ≥1 drink/month. Occupations were divided into five groups: unemployed (e.g., students, housewives); office workers (e.g., managers, professionals, and office workers); sales and services; machine fitting and simple labor (e.g., technicians, device, and machine operators, and low-level laborers); and agriculture, forestry, and fishery [29]. Household income levels were categorized into four groups according to quartiles. Educational level was divided into four groups: ≤6 years (elementary school), 7–9 years (middle school), 10–12 years (high school), and ≥13 years (university or college). Physical activity was categorized into the following: (1) walking was defined as walking activity for five or more days per week for at least 30 min; (2) moderate physical activity was defined as mid-intensity physical activity ≥ 5 days per week for at least 30 min; and (3) high physical activity was defined as high-intensity physical activity ≥ 3 days per week for at least 20 min. Depressive symptoms were defined as individuals who felt sad or had depressive symptoms for two consecutive weeks during the past year.

### 2.4. Statistical Analysis

Statistical analyses were performed using Stata/MP 15.0 (StataCorp., 2017, Stata Statistical Software: Release 15; College Station, TX, USA; StataCorp LP). Continuous variables are presented as mean ± standard deviation. Statistical significance was considered at a two-tailed *p*-value of < 0.05. Sampling weights were applied to the study population to represent the Korean population, without bias.

General demographics and co-variables were evaluated between participants with and without CLBP. The Student’s t-test was used to compare continuous variables, and the chi-square test was used for categorical variables. The baseline demographics and co-variables listed in Table 1 were assessed as independent variables for the models.

To create a development and validation dataset from the entire dataset, we used the split sample method [30]. A split sample with a 50% hold out results in models with suboptimal performance that are unstable and of the same performance as that obtained with half the sample size [31]. Therefore, the development dataset for the prediction equation was obtained by randomly selecting 80% of the entire dataset [32]. A logistic regression model was used to develop prediction equations. Only covariables with a *p*-value of <0.05 from univariate analysis were subsequently evaluated in the multiple logistic regression, using backward stepwise selection with a significance level of 0.05. Table 2 shows the list of variables, odds ratios, and regression coefficients that remained in the final prediction models after multiple logistic regression.

The developed model was validated by evaluating its performance with respect to discrimination and calibration using C-statistics and Hosmer–Lemeshow chi-square statistics. The validation datasets for the prediction equation consisted of the remaining 20% of the full dataset after the development dataset was randomly selected. The area under the receiver operating characteristic curve (AUC), also called the C-statistic, for the prediction model was measured for discrimination. Although it is controversial to determine the good value of the AUC, the AUC results were categorized into the following: suboptimal (<0.70), good (0.70–0.80), and excellent (≥0.80). To assess the developed model calibration, Hosmer–Lemeshow chi-square statistics (HLS) were used to calculate the closeness of the predicted risks to the actual observed risks. To calculate HLS, the dataset was divided into 10 subgroups based on the predicted probabilities from the developed prediction model. Values exceeding 20 indicate a significant lack of calibration [33].

The risk of CLBP was predicted using a nomogram, which is a two-dimensional diagram designed to allow approximate graphical computation of a mathematical function. The nomogram was generated using independent risk factors analyzed in multiple logistic regression analysis. To create the nomogram, we used the “nomolog” module of STATA [34].

### 2.5. Ethics Approval and Consent to Participate

The IV-1, IV-2, and IV-3 versions of the KNHANES were approved by the KCDC Institutional Review Board (approval no. 2007-02CON-04-P, 2008-04EXP-01-C, 2009-01CON-03-2C). Informed consent was obtained from all participants and their parents or guardians for those who were under 16 years of age when the surveys were conducted.

## 3. Results

### 3.1. Demographics of Participants

The baseline characteristics of all the participants are presented in Table 1. A total of 17,038 participants were analyzed, including 2693 with CLBP and 14,345 without CLBP. The prevalence of CLBP was 15.8% in the Korean participants, with a prevalence of 11.8% in men and 24.5% in women. Of these, 80% of the participants (*n* = 13,630) were randomly selected from the development dataset, and the remaining 20% of the participants (*n* = 3408) were selected for the validation dataset. In the development dataset, the number of participants with CLBP was 2120, and that without CLBP was 11,510. The mean age was 49.1 ± 16.6 years, and 5776 participants (42.4%) were men. In the validation dataset, there were 573 participants with CLBP and 2835 without CLBP. The mean age was 49.3 ± 16.6 years, and 1426 participants (41.8%) were men.

### 3.2. Risk Factors for the Prediction Model

Table 2 shows the estimated odds ratios (ORs) from multiple logistic regressions in the development dataset. The selected risk factors include age, sex, occupation, education level, mid-intensity physical activity, depressive symptoms, and comorbidities (stroke, ischemic heart disease, knee osteoarthritis, asthma, chronic obstructive pulmonary disease, and cancer history). The CLBP prediction equation was calculated based on this risk analysis and the coefficients of the risk factors were developed. From this equation, the linear function for developing the prediction probability was estimated.

### 3.3. Discrimination and Calibration of the Prediction Model

**Figure 2:**
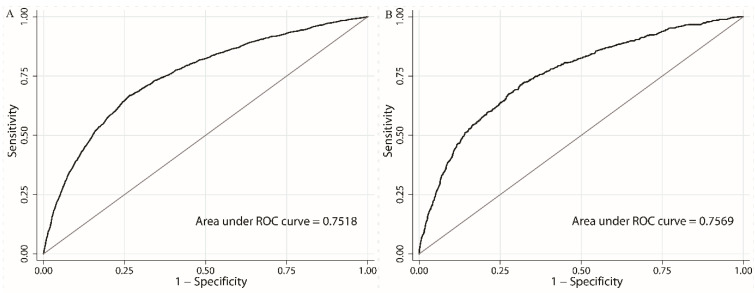
Discrimination plots in the (**A**) development and (**B**) validation datasets.

**Figure 3:**
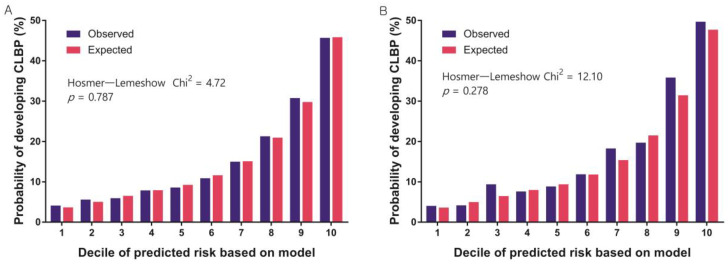
Calibration plots in the (**A**) development and (**B**) validation datasets.

Our prediction model had good discrimination (AUC = 0.7518) and was well-calibrated (HLS = 4.72, *p* = 0.787) in the development dataset. Moreover, this model showed good validation in the validation dataset (AUC = 0.7569, HLS = 12.10, *p* = 0.278). This indicates no significant differences between the observed and predicted probabilities according to our model. Figure 2 and Figure 3 show the discrimination and calibration plots of the CLBP prediction model.

### 3.4. Nomogram for the Prediction Model

**Figure 4:**
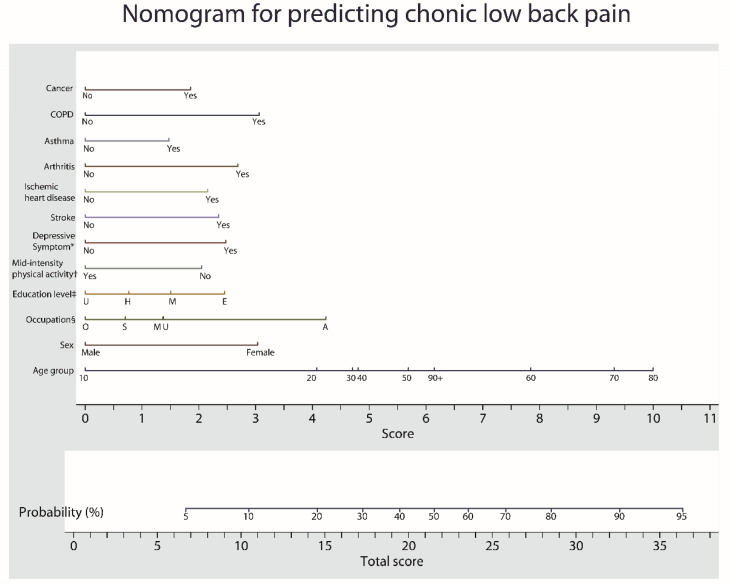
Nomogram for predicting chronic low back pain based on the risk factors. The value of each risk factor is respectively loaded on each variable axis (the 1st–12th lines), and a line is drawn downwards to determine the number of points received for each variable (the 13th line). Then, the sum of these numbers is located on the total points axis (the last line), and a line is drawn upwards to the risk probability axis to determine the likelihood of chronic low back pain. COPD, chronic obstructive pulmonary disease. * Depressive symptom was defined as individuals in this survey who felt sad or had a depressive symptom for two consecutive weeks during the past year. ^†^ Mid-intensity physical activity was defined as mid-intensity physical activity for 5 or more days per week for at least 30 min. ^‡^ U: ≥13 years (college or university), H: 10–12 years (high school), M: 7–9 years (middle school), E: ≤6 years (elementary school). ^§^ O: Office worker, S: Sales and services, U: Unemployed (student, housewife, etc.), M: Machine fitting and simple labor, A: Agriculture, forestry and fishery.

The risk factors that were found to predict CLBP in the development dataset were incorporated into the nomogram, as shown in Figure 4. The value of each risk factor is loaded on each variable axis (the 1st–12th lines), and a line is drawn downwards to determine the number of points received for each variable (the 13th line). The sum of these numbers is then located on the total point axis (the last line), and a line is drawn upward to the risk probability axis to determine the likelihood of CLBP.

## 4. Discussion

Our study developed and validated a clinical risk prediction model for CLBP in the general population using a cross-sectional Korean population-based health survey. It is necessary to create a risk prediction model to identify common risk factors of CLBP and to modify these risk factors, especially as there are increasing needs not only in the research setting but also in a clinical setting. To our knowledge, this is the first clinical risk-prediction model for CLBP in the general population. The prevalence of CLBP in the general population was 15.8% (11.8% in men and 24.5% in women). In the variables of demographics, medical history, and socioeconomic status, we identified significant risk factors that influenced the development of CLBP. Using these risk factors, we developed a clinical risk-prediction model and nomogram to allow personalized CLBP prediction based on personal characteristics. Our final model showed good discrimination and calibration performance in the validation datasets, which demonstrated the accurate prediction of CLBP in a new population with similar characteristics.

In our model, age was a key predictor of CLBP development. At 80 years of age, the odds ratio increased to 7.268, compared to 10 years of age (reference age). There are variations in prevalence depending on the study, but it is believed that it rises with age [9,35]. In accordance with previous studies, the risk of CLBP increased with age up to the 9th decade of life. The increased risks in women were similar to those found in a previous study [21]. However, our study revealed no significant difference between smokers and alcoholics when compared with non-smokers and non-alcoholics [36]. Other prognostic factors, such as occupation, education level, physical activity, depressive symptoms, and comorbidities, were associated with CLBP, which is similar to the findings of previous studies [10,11,12,24].

Previous studies have investigated the prognostic factors for developing CLBP from LBP in the workplace and general population. Heymans et al. reported higher pain intensity of initial LBP, no clinically relevant change in pain intensity, and disability status in the first three months; a higher score for kinesiophobia was most strongly related to CLBP in the workplace [25]. Similar studies in the general population also revealed that age, sex, height, health status, heavy work, chronic stress, decreased physical activity, smoking, and a history of LBP were important predictors of CLBP [35]. In a recent systematic review, several factors (such as anxiety, depression, generalized body pain, and leg pain) were identified to be directly associated with CLBP in the working population, whereas others were age-related (such as knee osteoarthritis or retirement due to ill health) [9]. Importantly, a moderate amount of leisure-time physical activity was identified as a protective factor against the development of CLBP in older adults [37]. There are some differences between these studies and our study. First, these studies have the advantage of prospectively detecting the development of CLBP, but they only analyzed individuals in the workplace or the general population with a small number of participants. Second, these studies only analyzed the prognostic factors that caused CLBP in patients with LBP. Although our study was not as prospective as previous studies, we analyzed the risk factors of patients with CLBP in nationwide representative populations with a relatively large population and analyzed the probability of developing CLBP. Multiple logistic regression was used to calculate this, and a nomogram was constructed using the coefficients. Although it is more convenient for many people to use a web-based calculator than a nomogram in actual usage, our study could not provide web-based calculation services because of the absence of a funding source, web developer, and servers.

As our risk prediction model is comprehensive and sophisticated, it is important to develop it to modify the risks of CLBP. Previous studies have simply informed patients with several risk factors that they belong to the high-risk group, this does not provide practical help in the clinical setting. Our model shows the scores according to the risk factors and predicted probability of developing LBP using a nomogram, which clearly presents and explains the risk factors and probabilities to patients and physicians. It is relevant for clinicians to recommend these preventive measures or treatment strategies to their patients. Thus, it may be used not only in primary care but also in healthcare centers for the general population.

### Strengths and Limitations of This Study

To the best of our knowledge, this is the first study to investigate the predicted probability of CLBP by using a nationwide representative Korean sample in the general population. The greatest strength of our study was the increased external validity of our findings using the KNHANES data. However, our study has a few limitations. Firstly, it was conducted through a national health and nutrition examination survey, which was designed as a cross-sectional study. Therefore, the actual development of CLBP could not be analyzed in this study. To assess the development of CLBP, a large prospective cohort study of the general population is needed. Unlike other prediction models (i.e., prediction of surgical site infection), constructing a prediction model for CLBP in a cohort is difficult. Furthermore, CLBP is closely associated with age, socioeconomic status, and comorbidities. These factors are well collected in our KNHANES dataset, which is not a cohort dataset but a cross-sectional dataset. These datasets cannot assess actual CLBP development but may be useful for constructing a prediction model using risk factors. Secondly, KNHANES was designed to minimize sampling errors by utilizing a clustered, multi-stage, and random sampling method. However, a selection bias may exist because of missing data. Participants were selected from our raw data to minimize selection bias, but missing data inevitably led to bias. Unlike other studies, such as cohort studies and clinical trials, the imputation of missing values is impossible in our dataset. Therefore, we excluded participants with missing data, which were necessary for the analysis. Thirdly, the simple survey of CLBP used in this study did not evaluate the severity, source, or duration of CLBP, which would be possible with instruments for measuring pain on a scale (e.g., a visual analog scale pain score). Fourthly, this study could not analyze some prognostic factors of CLBP such as previous episodes of LBP, the severity of pain, and disability. However, this study did analyze many other risk factors that have not been analyzed in other studies. Fifthly, the CLBP prediction model may be dependent on ethnicity. KNHANES is a health survey of the general Korean population. Therefore, this model is representative of the general Korean population, and caution is warranted when extrapolating these findings to other ethnic populations. Lastly, the data used in this study were collected from 2007 to 2009. The investigation of CLBP was conducted only at this time. A follow-up study will be conducted if additional data are available.

## 5. Conclusions

The risk of CLBP development can be reliably estimated in the general population. We developed a clinical risk prediction model to determine the probability of developing CLBP using a cross-sectional Korean population-based survey. Our prediction model showed good accuracy for both the development and validation datasets. Moreover, our risk prediction model, presented by a nomogram, which is a score-based prediction system, can be incorporated in a clinical setting. Thus, our prediction model with a nomogram can help individuals at risk of developing CLBP to receive appropriate counseling on risk modification from primary physicians.

## Figures and Tables

**Table 1:** Characteristics of the study population according to chronic low back pain.

Variables	Without CLBP (*n* = 14,345)	CLBP (*n* = 2693)	*p*-Value
Age, year	47.3 (± 16.1)	59.1 (± 16.0)	<0.001
Age, *n* (%)			
10–19	174 (1.2%)	4 (0.1%)	<0.001
20–29	1932 (13.5%)	142 (5.3%)	
30–39	3143 (21.9%)	269 (10.0%)	
40–49	3052 (21.3%)	310 (11.5%)	
50–59	2423 (16.9%)	439 (16.3%)	
60–69	2038 (14.2%)	698 (25.9%)	
70–79	1296 (9.0%)	665 (24.7%)	
80–89	275 (1.9%)	161 (6.0%)	
≥90	12 (0.1%)	5 (0.2%)	
Gender, *n* (%)			
Male	6444 (44.9%)	758 (28.1%)	<0.001
Female	7901 (55.1%)	1935 (71.9%)	
Height, cm	162.4 (± 9.1)	157.3 (± 9.3)	<0.001
Weight, kg	62.5 (± 11.5)	59.0 (± 10.3)	<0.001
BMI, kg/m^2^	23.6 (± 3.4)	23.8 (± 3.3)	0.002
Obesity, *n* (%) *			
Underweight (<18.5)	683 (4.8%)	106 (4.0%)	0.001
Normal (18.5–24.9)	9080 (64.1%)	1644 (61.6%)	
Obese (>25)	4403 (31.1%)	918 (34.4%)	
Smoking status, *n* (%)			
Non/Ex-smoker	11,018 (76.9%)	2304 (85.7%)	<0.001
Current smoker	3318 (23.1%)	274 (14.3%)	
Alcohol consumption, *n* (%)			
None	6453 (45.0%)	1688 (62.7%)	<0.001
≥1 drink/month	7892 (55.0%)	1005 (37.3%)	
Occupation, *n* (%)			
Unemployed (student, housewife, etc.)	5791 (40.4%)	1375 (51.1%)	<0.001
Office work	2891 (20.2%)	187 (6.9%)	
Sales and services	1893 (13.2%)	221 (8.2%)	
Agriculture, forestry and fishery	1102 (7.7%)	524 (19.5%)	
Machine fitting and simple labor	2668 (18.6%)	386 (14.3%)	
Household income, *n* (%) ^†^			
Low	2572 (18.4%)	976 (36.9%)	<0.001
Low-moderate	3510 (25.1%)	645 (24.4%)	
Moderate-high	3881 (27.8%)	554 (20.9%)	
High	4008 (28.7%)	471 (17.8%)	
Education level, *n* (%) ^‡^			
≤6 years	3462 (24.1%)	1503 (55.8%)	<0.001
7–9 years	1594 (11.1%)	316 (11.7%)	
10–12 years	5289 (36.9%)	563 (20.9%)	
≥13 years	4000 (27.9%)	311 (11.5%)	
Physical activity, *n* (%) ^§^			
Walk	6570 (46.0%)	1263 (47.1%)	0.32
Middle PA	1903 (13.3%)	514 (19.1%)	<0.001
High PA	2276 (15.9%)	408 (15.2%)	0.34
Depressive symptom, *n* (%) ^||^	1987 (13.9%)	698 (25.9%)	<0.001
Comorbidities, *n* (%)			
Hypertension	2591 (18.1%)	879 (32.6%)	<0.001
Dyslipidemia	968 (6.7%)	305 (11.3%)	<0.001
Stroke	261 (1.8%)	134 (5.0%)	<0.001
Ischemic heart disease	258 (1.8%)	133 (4.9%)	<0.001
Knee osteoarthritis	2119 (14.8%)	1052 (39.1%)	<0.001
Asthma	512 (3.6%)	215 (8.0%)	<0.001
COPD	114 (0.8%)	57 (2.1%)	<0.001
Diabetes	1004 (7.0%)	310 (11.5%)	<0.001
Chronic kidney disease	48 (0.3%)	17 (0.6%)	0.022
Liver cirrhosis	23 (0.2%)	10 (0.4%)	0.022
Cancer ^¶^	356 (2.5%)	145 (5.4%)	<0.001

Numeric parameters are expressed as mean and standard deviation in parentheses. Categorical parameters are expressed as counts and percentages in parentheses. CLBP, chronic low back pain; BMI, body mass index; PA, physical activity; COPD, chronic obstructive pulmonary disease. * Body mass index was categorized into underweight (<18.5 kg/m^2^), normal (18.5–24.9 kg/m^2^), and obese (≥25.0 kg/m^2^). ^†^ Household income level was calculated by dividing the total household monthly income with the obtained levels then grouped into quartiles. ^‡^ Educational level was divided into the following four groups: ≤6 years (elementary school), 7–9 years (middle school), 10–12 years (high school), and ≥13 years (college or university). ^§^ Physical activity was defined as three categories: walking was defined as walking activity for 5 or more days per week at least 30 min, middle physical activity was defined as mid-intensity physical activity for 5 or more days per week at least 30 min, and high physical activity was defined as high-intensity physical activity for 3 or more days per week at least 20 min. ^||^ Depressive symptom was defined as individuals in this survey who felt sad or had a depressive symptom for two consecutive weeks during the past one year. ^¶^ History of major cancer: stomach, liver, colon, breast, uterine cervical, prostate, or lung cancer.

**Table 2:** Chronic low back pain risk prediction model using multiple logistic regression in development dataset.

Variables	Coefficient	Odds Radio	95% CI		*p*-Value
Age group					
10–19	reference				
20–29	0.8090	2.246	0.810	6.224	0.120
30–39	0.9342	2.545	0.926	6.996	0.070
40–49	0.9534	2.595	0.945	7.122	0.064
50–59	1.1288	3.092	1.125	8.501	0.029
60–69	1.5562	4.741	1.722	13.049	0.003
70–79	1.8467	6.339	2.297	17.494	<0.001
80–89	1.9834	7.268	2.580	20.473	<0.001
≥90	1.2191	3.384	0.625	18.334	0.157
Gender					
Male	reference				
Female	0.6023	1.826	1.623	2.055	<0.001
Occupation					
Unemployed (Student, housewife, etc.)	reference				
Office work	−0.2717	0.762	0.620	0.937	0.010
Sales and services	−0.1310	0.877	0.730	1.054	0.161
Agriculture, forestry and fishery	0.5694	1.767	1.516	2.060	<0.001
Machine fitting and simple labor	0.0195	1.002	0.859	1.169	0.980
Education level *					
≤6 years	reference				
7–9 years	−0.1874	0.829	0.700	0.981	0.029
10–12 years	−0.3344	0.716	0.607	0.844	<0.001
≥13 years	−0.4863	0.615	0.499	0.757	<0.001
Middle PA ^†^	0.4069	1.502	1.315	1.716	<0.001
Depressive symptom ^‡^	0.4907	1.633	1.448	1.843	<0.001
Comorbidities					
Stroke	0.4657	1.593	1.223	2.075	0.001
Ischemic heart disease	0.4271	1.533	1.184	1.984	0.001
Knee osteoarthritis	0.5326	1.703	1.514	1.916	<0.001
Asthma	0.2921	1.339	1.090	1.646	0.005
COPD	0.6031	1.828	1.228	2.720	0.003
Cancer ^§^	0.3648	1.440	1.133	1.831	0.003

OR, Odds ratio; 95% CI, 95% confidence interval; PA, physical activity; COPD, chronic obstructive pulmonary disease. * Educational level was divided into the following four groups: ≤6 years (elementary school), 7–9 years (middle school), 10–12 years (high school), and ≥13 years (college or university). ^†^ Middle physical activity was defined as mid-intensity physical activity for 5 or more days per week at least 30 min. ^‡^ Depressive symptom was defined as individuals in this survey who felt sad or had a depressive symptom for two consecutive weeks during the past one year. ^§^ History of major cancer: stomach, liver, colon, breast, uterine cervical, prostate, or lung cancer. Risk prediction model was fully adjusted by all co-variables listed in the table.

## Data Availability

The data used in this study were sourced from the KCDC (Korea Centers for Disease Control & Prevention) in Korea. Owing to the legal restrictions imposed by the Government of Korea related to the Personal Information Protection Act, the dataset cannot be made publicly available. Interested researchers can obtain the dataset through a formal application to Division of Health and Nutrition Survey, KCDC, Korea (https://knhanes.cdc.go.kr/knhanes/eng/index.do) (accessed on 21 July 2019). Researchers are not allowed to carry raw data outside the KCDC. Interested researchers can access these data in the same manner as the authors. The authors did not have any special access privileges that other researchers would not have. The authors used 2007-2009 KNHANES data. All files are available from the KNHANES webpage (https://knhanes.cdc.go.kr/knhanes/sub03/sub03_02_02.do) (accessed on 21 July 2019) and the name of datasets (HN07_ALL.SAV, HN08_ALL.SAV, HN09_ALL.SAV) (accessed on 21 July 2019).
